# Intravenous Thrombolysis for Acute Ischemic Stroke in Patients with End-Stage Renal Disease on Hemodialysis: A Narrative Review

**DOI:** 10.3390/jcdd9120446

**Published:** 2022-12-09

**Authors:** Shuhei Egashira, Masatoshi Koga, Kazunori Toyoda

**Affiliations:** Department of Cerebrovascular Medicine, National Cerebral and Cardiovascular Center, 6-1 Kishibe-Shimmachi, Suita, Osaka 564-8565, Japan

**Keywords:** intravenous thrombolysis, acute ischemic stroke, end-stage renal disease, hemodialysis, activated partial thromboplastin time

## Abstract

Objectives: Acute ischemic stroke (AIS) is a significant and devastating complication in patients with end-stage renal disease on hemodialysis (ESRD/HD). Since one-third of AIS in ESRD/HD patients occurs during or soon after dialysis, patients are more likely to present within the time window when intravenous thrombolysis (IVT) can be performed. IVT may improve prognosis in ESRD/HD patients with AIS. However, ESRD/HD patients have been excluded from large trials and may have been withheld from IVT due to concerns about bleeding complications. To date, there is no clear evidence and firm guidance on the safety and efficacy of IVT in ESRD/HD patients with AIS. This narrative review aimed to evaluate critical scientific data on the benefits and risks of IVT use in patients with ESRD/HD and AIS. Materials and Methods: We searched the electronic database of PubMed for studies evaluating the relationship between AIS, ESRD/HD, and IVT. Reference sections and additional publications were also searched manually. Studies on AIS in patients with ESRD/HD requiring maintenance dialysis that referred to IVT were included. Results: In total, 560 studies were found in the PubMed electronic database during the period covered, of which 10 met the selection criteria. IVT for AIS in ESRD/HD patients could improve neurological outcomes and be safely performed even with the possibility of hemorrhagic complications associated with hypertension. Despite the high complication and mortality rates in ESRD/HD patients with AIS after IVT, the association with IVT was unclear. Conclusions: IVT for AIS in ESRD/HD patients may improve outcomes and should not be withheld based solely on ESRD/HD status.

## 1. Introduction

Stroke is the leading cause of death in patients with end-stage renal disease on hemodialysis (ESRD/HD); stroke mortality in ESRD/HD patients is nine times higher than in the general population [1,2]. The incidence of stroke is also 8–10 times higher in ESRD/HD patients than in the general population, with an annual incidence of acute ischemic stroke (AIS) reported being 4% [3,4,5]. One-third of AIS in ESRD/HD patients occurs during or within 30 min after dialysis, and patients regularly attend medical facilities, which makes the patients more likely to present within the time window in which intravenous thrombolysis (IVT) can be performed [6]. Although IVT may improve prognosis in ESRD/HD patients with AIS, the NINDS rt-PA trial and ECASS III, which first tested the efficacy and safety of IVT, excluded ESRD/HD patients [7,8]. Some recent randomized controlled trials comparing tenecteplase to alteplase also excluded ESRD/HD patients (NCT02814409). In addition, it has been suggested that reduced renal function may increase symptomatic intracranial bleeding, complications, and in-hospital mortality after IVT for AIS, leading some clinicians to refrain from performing IVT in ESRD/HD patients [9,10,11,12]. To date, there is no clear evidence and firm guidance on the safety and efficacy of IVT in ESRD/HD patients with AIS.

This narrative review aimed to evaluate critical scientific data on the benefits and risks of IVT use in patients with ESRD/HD and AIS.

## 2. Materials and Methods

Studies evaluating the relationship between ESRD/HD, AIS, and IVT were searched in the PubMed electronic database from January 2000 to October 2022. Three term groups were used in the search. The first term group is “end stage renal failure ^ end stage renal disease ^ hemodialysis ^ renal dysfunction ^ renal function”, the second group is “thrombolytics ^ fibrinolytic ^ thrombolysis ^ fibrinolysis”, and the third group is “stroke ^ acute ischemic stroke ^ AIS”. OR searches were performed within a term group, and AND searches were conducted between three-term groups. Reference sections and additional publications were also searched manually. Studies on AIS in patients with ESRD/HD requiring maintenance dialysis that referred to IVT were included. Critical data (study design, general characteristics of the study, sample size, and results on the safety and efficacy) were extracted from the included studies.

## 3. Results

The search using the above terms resulted in 560 studies found in the PubMed electronic database during the period covered, of which 10 met the selection criteria. Table 1 shows the details of each study. No randomized clinical trials were testing IVT in patients with ESRD/HD.

## 4. Discussion

### 4.1. Descriptive Cases of IVT for AIS in ESRD/HD Patients

Detailed descriptive cases of IVT for AIS in ESRD/HD were reported from Japan. Most concluded that IVT for AIS in ESRD/HD patients could improve neurological outcomes and be safely performed even with the possibility of hemorrhagic complications. Han et al. [13] reported a case report of an AIS patient with ESRD/HD who received IVT and achieved neurological improvement without complications. Naganuma et al. [14] described ESRD/HD patients selected from the Stroke Acute Management with Urgent Risk-factor Assessment and Improvement (SAMURAI) rt-PA registry, a multicenter observational study of alteplase use in Japan. Of the 600 AIS patients who received IVT, four (0.7%) had ESRD/HD. Of the four patients (three male, age 64–77, duration of dialysis 1.2–28 years), three (75%) had a stroke on the day of dialysis, one (25%) had a stroke during dialysis, and two (50%) had a stroke immediately after dialysis. One patient (25%) required intravenous antihypertensive therapy before IVT and had an ectopic cortical hematoma and intraventricular hemorrhage after IVT. The remaining three patients (75%) had no hemorrhagic complications. The modified Rankin scale score at three months was 0 in one patient (25%), 2 in two patients (50%), and 4 in one patient (25%). They concluded that AIS patients with ESRD/HD may have an improved neurological prognosis, although they are at risk for IVT-induced intracranial bleeding associated with hypertension. However, these reports are based on the Japanese recommended dose of 0.6 mg/kg alteplase, and the results may differ from the internationally used dose of 0.9 mg/kg [23,24,25].

### 4.2. The Time Window for IVT

Regarding the time window for IVT, some reports indicate that ESRD/HD patients are more likely to develop AIS in environments with good access to IVT facilities. Others, however, pointed out that ESRD/HD patients may be less likely to receive IVT promptly versus non-ESRD/HD patients. Cherian L et al. [15] retrospectively analyzed data from 34 ESRD/HD patients with AIS and transient ischemic attack at Rush University Medical Center by chart review. In that case series, 9 of 34 patients (27%) presented within the IVT time window, of whom 4 (44%) reported receiving IVT. Cohen-Nagai et al. [16] retrospectively studied stroke cases in ESRD/HD patients at a single institution. They said that all ESRD/HD patients developed AIS during hospitalization or dialysis, which allowed for prompt neurological evaluation, imaging, and IVT administration, resulting in neurological improvement and no complications. On the other hand, Findalay et al. [17] examined the inequality of care for strokes in ESRD/HD patients using a retrospective national data linkage cohort. He found that ESRD/HD patients were significantly less likely to have IVT performed within 60 min versus non-ESRD/HD patients (1.5% versus 3.9%; *p* = 0.016). The delay or withholding of IVT for ESRD/HD patients, despite their potentially better access to IVT, may be due to avoidance by providers of high-risk cases and difficulties in blood pressure control, as well as the problem of prolonged activated partial thromboplastin time (APTT), which will be discussed later.

### 4.3. Complications and in-Hospital Mortality, Management in the Stroke Care Unit after IVT

Given the relatively poor outcomes after IVT for AIS in patients with chronic kidney disease, complications and mortality after IVT for AIS in ESRD/HD patients were studied using the National Inpatient Sample in the United States [9,10,11,18,19]. Despite the high complication and mortality rates in ESRD/HD patients with AIS after IVT, the association with IVT was unclear, and reports suggested that IVT should not be withheld simply because a patient has ESRD/HD. According to Tariq N et al. [18], there was no significant difference in the incidence of intracranial hemorrhage with or without ESRD/HD (5.2% versus 6.1%). The results are not adjusted for relevant factors and may be subject to patient selection bias. ESRD/HD patients had more complications during hospitalization, such as pneumonia, deep vein thrombosis, and sepsis, and significantly higher in-hospital mortality than non-ESRD/HD patients (22% versus 11%, *p* ≤ 0.0001). However, among AIS patients who did not receive IVT, dialysis patients had higher in-hospital mortality rates than non-dialysis patients, and it is unclear whether IVT contributed to the increase in in-hospital mortality. Pana TA et al. [19] also conducted a retrospective comparative study using the National Inpatient Sample. They noted that compared with the no-chronic kidney disease group, the ESRD/HD groups had significantly increased odds of in-hospital mortality (odds ratio (99% confidence interval); 2.06, (1.90–2.25), *p* < 0.001), were at higher odds of prolonged hospitalization (1.44, (1.37–1.51), *p* < 0.001), and were at higher odds of moderate-to-severe disability on discharge (1.13 (1.10–1.15), *p* < 0.001) after IVT. Interaction terms between IVT and the ESRD/HD groups were not statistically significant (*p* > 0.01) for any outcome. They concluded that IVT should not be withheld for AIS patients with ESRD. Power A et al. [20] also noted in their review article that IVT can be safely performed for AIS, although its efficacy may be attenuated in patients with chronic renal failure and ESRD. Although in-hospital complications and mortality were problems in these studies, management in the stroke care unit (SCU) has generally been shown to reduce complications and mortality in stroke patients [26,27,28,29,30]. Findalay et al. [17] reported that management in the SCU also reduced mortality in ESRD/HD patients who developed AIS; SCU management may be preferable for AIS patients with ESRD/HD receiving IVT.

### 4.4. Expert Opinion

In the expert opinion survey, the prevailing view was that IVT of AIS in ESRD/HD patients should not be refrained from as long as possible. In an opinion survey of US stroke experts, 33% of respondents had used intravenous thrombolysis, and most experts would use it when it was feasible [21]. In a survey of nephrologists, 75% preferred to be involved in IVT decision-making due to concerns about bleeding complications. While multi-specialist consultation is desirable, making decisions quickly remains challenging [22].

### 4.5. Practical Issues: Stroke Subtype

To date, there is no evidence of the efficacy of IVT in ESRD/HD patients by stroke subtypes. K.T. et al. reported stroke subtypes of 57 AIS patients with ESRD/HD in a multicenter, retrospective study: small vessel occlusion in 48%, large artery atherosclerosis in 27%, and cardioembolism in 25% [6]. Jung S et al. also reported stroke subtypes at a single center: small vessel occlusion in 48%, large artery atherosclerosis in 28%, undetermined etiology in 24%, and cardioembolism in 1% [31]. In both reports, small vessel occlusion was the most common stroke subtype of AIS in ESRD/HD patients. However, small vessel occlusion was not included among the subtypes of five cases from the case report or case series in this review, in which stroke subtype data were available. Three of the five patients had cardioembolism, and two had undetermined etiology [13,14]. This dissociation may be accounted for by selection bias. Small vessel occlusion is the stroke subtype with the best functional prognosis [32]. Therefore, when ESRD/HD patients develop small vessel occlusion, IVT may have been withheld because of the limited benefit of the prognosis. The balance of benefits and risks of IVT according to stroke subtypes needs to be carefully considered and further investigated.

### 4.6. Practical Issues: Stroke Severity

There is no evidence that IVT for AIS in ESRD/HD patients should be selected according to stroke severity. In the case report and case series included in this review with available baseline National Institute of Health Stroke Scale (NIHSS) score data, stroke severity was distributed from mild to severe, with NIHSS scores ranging from 4 to 20 [13,14]. The higher the stroke severity, the higher the risk of hemorrhage after IVT [33,34]. However, only one patient with hemorrhagic complications had the lowest NIHSS score of 4, suggesting that IVT for ESRD/HD patients can improve neurological symptoms for severe stroke but can cause hemorrhagic complications even in patients with mild stroke. These results may also be subject to selection bias and influenced by the stroke subtypes described above.

### 4.7. Practical Issues: Stroke Lesion and Occluded Vessel

There are no studies on stroke lesions and occluded vessels in AIS patients with ESRD/HD who underwent IVT. According to K.T. et al., 57% of stroke lesions in ESRD/HD patients were in the anterior circulation and 43% in the posterior circulation [6]. Jung S et al. found that the anterior circulation accounted for 68%, the posterior circulation for 24%, and both for 8% [31]. In the five cases in this review for which data on stroke lesions and occluded vessels were available, all were from the anterior circulation, but none were from the posterior circulation. The occluded vessels were identifiable in three cases, and the intracranial carotid artery, M1, and M2 segment of the middle cerebral artery were identified in one patient each [13,14]. There were no data on AIS of the posterior circulation or in cases with tandem occlusion of intracranial and cervical vessels. The efficacy of IVT for AIS in the posterior circulation and each occluded vessel needs further investigation.

### 4.8. Practical Issues: Prolonged APTT

“A 65-year-old hemodialysis patient with diabetes mellitus, hypertension, and atrial fibrillation presented to the hospital 1 h after the sudden onset of aphasia and right-sided hemiparalysis immediately after the end of hemodialysis (4-h dialysis) in which he received a total of 5000 units of heparin. He had an NIHSS score of 18, blood pressure of 160/105 mmHg, and no findings on non-contrast CT of the head. He is eligible for IVT except for a moderately prolonged APTT of 47 s (baseline is 35 s). Should this patient be treated with intravenous thrombolysis?”

Because unfractionated heparin is often used during hemodialysis, prolonged APTT can be a practical clinical issue when considering IVT for AIS in ESRD/HD patients. The case presented in the 2011 US expert opinion survey, partially modified, is the one at the beginning of this chapter [21]. About half of the US experts (17 out of 40) responded that even if the guidelines were violated, they would immediately administer IVT because “time is brain”. They would consider withholding IVT only when the APTT was 1.5 times higher than the baseline. In the same case, 80% of respondents indicated that they would repeat the APTT without waiting 2 h, which was agreed upon by 11 European experts [21]. Protamine should not be used in hyperacute stroke patients because it can increase coagulation and worsen cerebral circulation [35]. When the APTT is prolonged, IVT can be administered without contraindication if the patient is still within the time window after retesting and correction of the prolongation, but the validity of delaying IVT to normalize the APTT is not clear. In recent years, several randomized clinical trials have reported skipping IVT for AIS with large vessel occlusion when mechanical thrombectomy is feasible [36,37,38,39]. The potential treatment options for the case mentioned above are as follows: “Perform IVT immediately because the APTT value is not more than 1.5 times the previous value”, “Skip IVT and consider mechanical thrombectomy”, and “Consider IVT when the APTT value reaches about 40 s after some time has elapsed”. All of these are appropriate and should be considered according to the policies and conditions of each institution. On the other hand, unfractionated heparin used for hemodialysis is metabolized relatively quickly. ESRD/HD patients with prolonged APTT on the presentation for AIS are rare [40,41]. Further investigation is needed for IVT in hemodialysis patients with prolonged APTT.

### 4.9. Practical Issues: Atherosclerosis and Atrial Fibrillation

Patients with ESRD/HD are more likely to have coexisting advanced atherosclerotic lesions [6,14,15,34]. ESRD/HD patients also have a high rate of atrial fibrillation (11–13%). ESRD/HD patients with severe atherosclerosis often have large vessel occlusion due to cardioembolism and are candidates for revascularization therapy [42,43,44]. When thrombectomy is attempted, the femoral artery or aortic arch may have severe atherosclerosis, making it difficult to secure an access route, or the carotid artery may be highly tortuous, making catheter insertion difficult. It is also known that bending tortuosity due to arteriosclerosis makes thrombectomy difficult and reduces its outcome [45]. Skipping IVT just because the patient has ESRD/HD is not recommended because it means giving up a potentially effective treatment option.

## 5. Limitations

The main limitation of this narrative review is its lack of accuracy and reproducibility, and we present our findings in the context of what has been reported. However, this may be an inherent limitation of narrative reviews. Most of the articles included in this review are retrospective, and patient selection bias must be considered regarding the efficacy and safety of IVT for AIS patients with ESRD/HD.

## 6. Conclusions

IVT for AIS in ESRD/HD patients may improve outcomes and should not be withheld based solely on ESRD/HD status.

Future Directions: There is no clear evidence on whether bleeding complications are more common in ESRD/HD patients after IVT for AIS. Data from the National Inpatient Sample in the US suggest no difference between patients and those not on dialysis. However, this is not adjusted for relevant factors and may be due to patient selection bias [18]. The differences in the efficacy of IVT for AIS patients with ESRD/HD by stroke subtype, severity, lesion, and occluded vessel have not been thoroughly investigated. There is no consensus on IVT in AIS patients with moderately prolonged APTT and ESRD/HD. Several randomized clinical trials have investigated skipping IVT before thrombectomy, which is essential in ESRD/HD patients who are assumed to be at high risk for bleeding. Still, it is unclear whether these results apply to ESRD/HD patients [36,37,38,39].

## Figures and Tables

**Table 1:** Summary of studies included.

Author	Published Year	Study Type	Intravenous Thrombolytic Agent Used and Dose	Total Population	The Proportion of ESRD/HD Patients Receiving IVT for AIS	Results of AIS Patients with ESRD on Dialysis Receiving IVT
Han W et al. [13]	2018	Case report	Alteplase (0.6 mg/kg)	1	One patient	A patient with ESRD/HD treated with IVT for AIS achieved neurological improvement without complications
Naganuma M et al. [14]	2011	Case series —The retrospective cohort of AIS patients receiving IVT from the SAMURAI rt-PA Registry	Alteplase (0.6 mg/kg)	600 AIS patients receiving IVT	Four patients with ESRD/HD (0.7%)	One patient (25%) developed an intracranial hemorrhage after IVT. The other three patients (75%) did not develop hemorrhagic complications. The modified Rankin Scale score at three months was 0 in one patient (25%), 2 in two patients (50%), and 4 in one patient (25%).
Cherian L et al. [15]	2015	Case series —ICD-9 codes were used to identify AIS and TIA patients with ESRD/HD admitted to the stroke service of Rush University Medical Center from 22 August 2011 to 21 June 2014.	No data	34 ESRD/HD patients with TIA/AIS	Four patients (11.8%)	Nine patients (26.5%) presented within the IVT time window, and four (11.8%) were eligible and received IVT.
Cohen-Hagai K et al. [16]	2019	Observational study —Retrospective, observational, case-control cohort study, the electronic medical records of all chronic hemodialysis patients at Meir Medical Center, Israel, from 1 January 2014 to 31 December 2017	No data	585 ESRD/HD patients on dialysis −52 (8.9%) with AIS	Four patients (7.7%)	ESRD/HD patients received a neurological evaluation, CT imaging, and TPA administration performed promptly because they developed AIS during hospitalization or dialysis, and neurological symptoms of stroke onset were confirmed. The neurological symptoms resolved after IVT. IVT for AIS patients with ESRD/HD was safe, and no bleeding was observed in these patients.
Findlay MD et al. [17]	2018	Observational study —Retrospective national data linkage cohort using data within the Scottish Renal Registry, Scottish Stroke Care Audit, Scottish MorbidIVTy Records, and the National Records of Scotland	No data	8757 ESRD/HD patients −224 ESRD/HD patients experienced a stroke	Three patients (1.5%) of AIS patients whose data were available)	ESRD/HD patients were less likely to receive IVT within 60 min than non-ESRD/HD patients (1.5% versus 3.9%; *p* = 0.016)
Tariq N et al. [18]	2013	Observational study —The retrospective cohort of AIS patients receiving IVT from the US National Inpatient Sample (2002–2009))	No data	82,142 AIS patients received IVT	1072 patients (1.3%) with ESRD/HD	Intracranial hemorrhage rates did not differ significantly between patients with ischemic stroke with or without dialysis who received thrombolytics (5.2% versus 6.1%). The in-hospital mortality rate was higher in dialysis-dependent patients treated with thrombolytics (22% versus 11%, *p* ≤ 0.0001). After adjusting for age, sex, and comorbidities, dialysis dependence was associated with higher rates of in-hospital mortality in patients treated with thrombolytics (odds ratio, 1.92; 95% confidence interval, 1.33–2.78, *p* = 0.0005).
Pana TA et al. [19]	2021	Observational study —The retrospective cohort of AIS patients receiving IVT from the US National Inpatient Sample (2005–2015)	No data	4,283,086 AIS patients −74,499 (1.7%) with ESRD/HD	2757 patients (3.7%)	Compared with the no CKD group, the ESRD/HD groups had significantly increased odds of in-hospital mortality (odds ratio (99% confidence interval); 2.06, (1.90–2.25), *p* < 0.001), were at higher odds of prolonged hospitalization (1.44, (1.37–1.51), *p* < 0.001), and were at higher odds of moderate-to-severe disability on discharge (1.13 (1.10–1.15), *p* < 0.001) after IVT. Interaction terms between IVT and the ESRD/HD groups were not statistically significant (*p* > 0.01) for any outcome.
Power A et al. [20]	2013	Narrative review about the association between CKD, ESRD, and stroke	No data	-	-	IVT for AIS appears safe even in all stages of CKD, although the therapeutic effect may be attenuated.
Palacio S et al. [21]	2011	Expert opinion —Sixty-five stroke experts in IVT of AIS were queried regarding their personal experience in the use of IVT in ESRD/HD patients. Hypothetical case scenarios were presented.	No data	-	-	Of the 65 stroke experts who were queried, 40 (62%) responded. One-third of the responders had previously treated ESRD/HD patients with IVT. Most favored use of IVT for ESRD/HD patients with AIS. When presented with a case of a patient who had recently undergone dialysis with a mildly prolonged activated partial thromboplastin time, 50% favored immediate IVT. Seventy-eight percent of the experts would have considered an intra-arterial approach and would have preferred mechanical clot retrieval to thrombolysis.
Power A et al. [22]	2013	Expert opinion —Consultant nephrologists participated in an internet-based questionnaire. Respondents were asked about their involvement in IVT decisions and safety concerns in ESRD patients.	No data	-	-	In total, 122/433 (28%) clinicians responded; 75% wanted involvement in thrombolysis decisions, although just 10% gave input in practice; 64% expressed a high degree of concern (≥7/10) regarding intracranial bleeding risk in hemodialysis. Overall, intra- and extracranial bleeding risks were lower in peritoneal dialysis (*p* < 0.001).

ESRD, end-stage renal disease; HD, hemodialysis; ESRD/HD, end-stage renal failure on hemodialysis; CKD, chronic kidney disease; AIS, acute ischemic stroke; IVT, intravenous thrombolysis; TIA, transient ischemic attack.

## Nonstandard Abbreviations and Acronyms

AIS	acute ischemic stroke
APTT	activated partial thromboplastin time
ESRD	end-stage renal disease
ESRD/HD	end-stage renal disease on hemodialysis
IVT	intravenous thrombolysis
SCU	stroke care unit
NIHSS	National Institute of Health Stroke Scale

## References

[1] De Jager D.J., Grootendorst D.C., Jager K.J., van Dijk P.C., Tomas L.M.J., Ansell D., Collart F., Finne P., Heaf J.G., De Meester J. (2009). Cardiovascular and noncardiovascular mortality among patients starting dialysis. JAMA.

[2] Ocak G., van Stralen K.J., Rosendaal F.R., Verduijn M., Ravani P., Palsson R., Leivestad T., Hoitsma A.J., Ferrer-Alamar M., Finne P. (2012). Mortality due to pulmonary embolism, myocardial infarction, and stroke among incident dialysis patients. J. Thromb. Haemost..

[3] Sozio S.M., Armstrong P.A., Coresh J., Jaar B.G., Fink N.E., Plantinga L.C., Powe N.R., Parekh R.S. (2009). Cerebrovascular disease incidence, characteristics, and outcomes in patients initiating dialysis: The choices for healthy outcomes in caring for ESRD (CHOICE) study. Am. J. Kidney Dis..

[4] Foley R.N., Gilbertson D.T., Murray T., Collins A.J. (2011). Long interdialytic interval and mortality among patients receiving hemodialysis. N. Engl. J. Med..

[5] Power A., Chan K., Singh S.K., Taube D., Duncan N. (2012). Appraising stroke risk in maintenance hemodialysis patients: A large single-center cohort study. Am. J. Kidney Dis..

[6] Toyoda K., Fujii K., Fujimi S., Kumai Y., Tsuchimochi H., Ibayashi S., Iida M. (2005). Stroke in patients on maintenance hemodialysis: A 22-year single-center study. Am. J. Kidney Dis..

[7] National Institute of Neurological Disorders and Stroke rt-PA Stroke Study Group (1995). Tissue plasminogen activator for acute ischemic stroke. N. Engl. J. Med..

[8] Bluhmki E., Chamorro A., Dávalos A., Machinig T., Sauce C., Wahlgren N., Wardlaw J., Hacke W. (2009). Stroke treatment with Alteplase given 3.0-4.5 h after onset of acute ischaemic stroke (ECASS III): Additional outcomes and subgroup analysis of a randomized controlled trial. Lancet Neurol..

[9] Naganuma M., Koga M., Shiokawa Y., Nakagawara J., Furui E., Kimura K., Yamagami H., Okada Y., Hasegawa Y., Kario K. (2011). Reduced estimated glomerular filtration rate is associated with stroke outcome after intravenous rt-PA: The Stroke Acute Management with Urgent Risk-Factor Assessment and Improvement (SAMURAI) rt-PA registry. Cerebrovasc. Dis..

[10] Lyrer P.A., Fluri F., Gisler D., Papa S., Hatz F., Engelter S.T. (2008). Renal function and outcome among stroke patients treated with IV thrombolysis. Neurology.

[11] Agrawal V., Rai B., Fellows J., McCullough P.A. (2010). In-hospital outcomes with thrombolytic therapy in patients with renal dysfunction presenting with acute ischaemic stroke. Nephrol. Dial. Transplant..

[12] De Los Rios F., Kleindorfer D.O., Guzik A., Ortega-Gutierrez S., Sangha N., Kumar G., Grotta J.C., Lee J.-M., Meyer B.C., Schwamm L.H. (2014). Intravenous fibrinolysis eligibility: A survey of stroke clinicians’ practice patterns and review of the literature. J. Stroke Cerebrovasc. Dis..

[13] Han W., Sakurada T., Hachisuka R., Kuroya S., Sumi H., Kojima S., Shibagaki Y., Tsuchihashi Y., Isahaya K., Sasaki N. (2018). A case of cerebral infarction during a hemodialysis procedure successfully treated with recombinant tissue plasminogen activator. CEN Case Rep..

[14] Naganuma M., Mori M., Nezu T., Makihara N., Koga M., Okada Y., Minematsu K., Toyoda K. (2011). Intravenous recombinant tissue plasminogen activator therapy for stroke patients receiving maintenance hemodialysis: The Stroke Acute Management with Urgent Risk-Factor Assessment and Improvement (SAMURAI) rt-PA registry. Eur. Neurol..

[15] Cherian L., Conners J., Cutting S., Lee V.H., Song S. (2015). Periprocedural Risk of Stroke Is Elevated in Patients with End-Stage Renal Disease on Hemodialysis. Cerebrovasc. Dis. Extra..

[16] Cohen-Hagai K., Nacasch N., Rozenberg I., Korzets Z., Einbinder Y., Zitman-Gal T., Benchetrit S. (2019). Clinical outcomes of stroke in hemodialysis patients: A retrospective single-center study. Int. Urol. Nephrol..

[17] Findlay M.D., Dawson J., MacIsaac R., Jardine A.G., Macleod M.J., Metcalfe W., Traynor J.P., Mark P. (2018). Inequality in Care and Differences in Outcome Following Stroke in People With ESRD. Kidney Int. Rep..

[18] Tariq N., Adil M.M., Saeed F., Chaudhry S.A., Qureshi A.I. (2013). Outcomes of thrombolytic treatment for acute ischemic stroke in dialysis-dependent patients in the United States. J. Stroke Cerebrovasc. Dis..

[19] Pana T.A., Quinn J., Mohamed M.O., Mamas M.A., Myint P.K. (2021). Thrombolysis in acute ischaemic stroke patients with chronic kidney disease. Acta Neurol. Scand..

[20] Power A. (2013). Stroke in dialysis and chronic kidney disease. Blood Purif..

[21] Palacio S., Gonzales N.R., Sangha N.S., Birnbaum L.A., Hart R.G. (2011). Thrombolysis for acute stroke in hemodialysis: International survey of expert opinion. Clin. J. Am. Soc. Nephrol..

[22] Power A., Fogarty D., Wheeler D.C. (2013). Acute stroke thrombolysis in end-stage renal disease: A national survey of nephrologist opinion. Nephron Clin. Pract..

[23] Mori E., Yoneda Y., Tabuchi M., Yoshida T., Ohkawa S., Ohsumi Y., Kitano K., Tsutsumi A., Yamadori A. (1992). Intravenous recombinant tissue plasminogen activator in acute carotid artery territory stroke. Neurology.

[24] Yamaguchi T., Hayakawa T., Kiuchi H. (1993). Intravenous Tissue Plasminogen Activator Ameliorates the Outcome of Hyperacute Embolic Stroke. Cerebrovasc. Dis..

[25] Yamaguchi T., Mori E., Minematsu K., Nakagawara J., Hashi K., Saito I., Shinohara Y. (2006). Alteplase at 0.6 mg/kg for acute ischemic stroke within 3 hours of onset: Japan Alteplase Clinical Trial (J-ACT). Stroke.

[26] Govan L., Langhorne P., Weir C.J., Stroke Unit Trialists Collaboration (2007). Does the prevention of complications explain the survival benefit of organized inpatient (stroke unit) care? Further analysis of a systematic review. Stroke.

[27] Stroke Unit Trialists Collaboration (1997). How do stroke units improve patient outcomes? A collaborative systematic review of the randomized trials. Stroke.

[28] Zhu H.F., Newcommon N.N., Cooper M.E., Green T.L., Seal B., Klein G., Weir N.U., Coutts S.B., Watson T., Barber P.A. (2009). Impact of a stroke unit on length of hospital stay and in-hospital case fatality. Stroke.

[29] Stroke Unit Trialists Collaboration (1997). Collaborative systematic review of the randomised trials of organised inpatient (stroke unit) care after stroke. BMJ.

[30] Pilato F., Silva S., Valente I., Distefano M., Broccolini A., Brunetti V., Caliandro P., Marca G.D., Di Iorio R., Frisullo F. (2020). Predicting Factors of Functional Outcome in Patients with Acute Ischemic Stroke Admitted to Neuro-Intensive Care Unit-A Prospective Cohort Study. Brain Sci..

[31] Jung S., Kwon S.B., Hwang S.H., Noh J.W., Lee Y.K. (2012). Ischemic stroke among the patients with end-stage renal disease who were undergoing maintenance dialysis. Yonsei Med. J..

[32] Rudilosso S., Rodríguez-Vázquez A., Urra X., Arboix A. (2022). The Potential Impact of Neuroimaging and Translational Research on the Clinical Management of Lacunar Stroke. Int. J. Mol. Sci..

[33] Weng Z.A., Huang X.X., Deng D., Yang Z., Li S., Zang J., Li Y.F., Liu Y.-F., Wu Y.-S., Zhang T.-Y. (2022). A New Nomogram for Predicting the Risk of Intracranial Hemorrhage in Acute Ischemic Stroke Patients After Intravenous Thrombolysis. Front. Neurol..

[34] Guo H., Xu W., Zhang X., Zhang S., Dai Z., Li S., Xie Y., Li Y., Xue J., Liu X. (2021). A Nomogram to Predict Symptomatic Intracranial Hemorrhage After Intravenous Thrombolysis in Chinese Patients. Neuropsychiatr. Dis. Treat..

[35] Toyoda K., Koga M., Iguchi Y., Itabashi R., Inoue M., Okada Y., Ogasawara K., Tsujino A., Hasegawa Y., Hatano T. (2019). Guidelines for Intravenous Thrombolysis (Recombinant Tissue-type Plasminogen Activator), the Third Edition, March 2019: A Guideline from the Japan Stroke Society. Neurol. Med. Chir..

[36] Suzuki K., Matsumaru Y., Takeuchi M., Morimoto M., Kanazawa R., Takayama Y., Kamiya Y., Shigeta K., Okubo S., Hayakawa M. (2021). Effect of Mechanical Thrombectomy Without vs With Intravenous Thrombolysis on Functional Outcome Among Patients With Acute Ischemic Stroke: The SKIP Randomized Clinical Trial. JAMA.

[37] Yang P., Zhang Y., Zhang L., Zhang Y., Treurniet K.M., Chen W., Peng Y., Han H., Wang J., Wang S. (2020). Endovascular Thrombectomy with or without Intravenous Alteplase in Acute Stroke. N. Engl. J. Med..

[38] Zi W., Qiu Z., Li F., Sang H., Wu D., Luo W., Liu S., Yuan J., Song J., Shi Z. (2021). Effect of Endovascular Treatment Alone vs Intravenous Alteplase Plus Endovascular Treatment on Functional Independence in Patients With Acute Ischemic Stroke: The DEVT Randomized Clinical Trial. JAMA.

[39] LeCouffe N.E., Kappelhof M., Treurniet K.M., Rinkel L.A., Bruggeman A.E., Berkhemer O.A., Wolff L., van Voorst H., Tolhuisen M.L., Dippel D.W. (2021). A Randomized Trial of Intravenous Alteplase before Endovascular Treatment for Stroke. N. Engl. J. Med..

[40] Brunet P., Simon N., Opris A., Faure V., Lorec-Penet A.M., Portugal H., Dussol B., Berland Y. (2008). Pharmacodynamics of unfractionated heparin during and after a hemodialysis session. Am. J. Kidney Dis..

[41] Meretoja A., Putaala J., Tatlisumak T., Atula S., Artto V., Curtze S., Häppölä O., Lindsberg P.J., Mustanoja S., Piironen K. (2010). Off-label thrombolysis is not associated with poor outcome in patients with stroke. Stroke..

[42] Vazquez E., Sanchez-Perales C., Garcia-Garcia F., Castellano P., Garcia-Cortes M.-J., Liebana A., Lozano C. (2009). Atrial fibrillation in incident dialysis patients. Kidney Int..

[43] Wizemann V., Tong L., Satayathum S., Disney A., Akiba T., Fissell R.B., Kerr P.G., Young E.W., Robinson B.M. (2010). Atrial fibrillation in hemodialysis patients: Clinical features and associations with anticoagulant therapy. Kidney Int..

[44] Zimmerman D., Sood M.M., Rigatto C., Holden R.M., Hiremath S., Clase C.M. (2012). Systematic review and meta-analysis of incidence, prevalence and outcomes of atrial fibrillation in patients on dialysis. Nephrol. Dial. Transplant..

[45] Koge J., Tanaka K., Yoshimoto T., Shiozawa M., Kushi Y., Ohta T., Satow T., Kataoka H., Ihara M., Koga M. (2022). Internal Carotid Artery Tortuosity: Impact on Mechanical Thrombectomy. Stroke..

